# Deregulated expression of microRNA-200b/c and SUZ12, a Polycomb repressive complex 2 subunit, in chemoresistant colorectal cancer cells

**DOI:** 10.18632/genesandcancer.152

**Published:** 2017-07

**Authors:** KayKay San, Megan Horita, Aravinda Ganapathy, G. Chinnadurai, Uthayashanker R. Ezekiel

**Affiliations:** ^1^ Department of Clinical Health Sciences, Doisy College of Health Sciences, Saint Louis University, St. Louis, MO, USA; ^2^ Institute for Molecular Virology and Department of Molecular Microbiology and Immunology, Doisy Research Center, School of Medicine, Saint Louis University, St. Louis, MO, USA

**Keywords:** SUZ12, miR-200, colorectal cancer, chemoresistance, oxaliplatin

## Abstract

In colorectal cancer, chemotherapy and/or radiotherapy can lead to the formation of resistant cells that become metastatic through Epithelial-Mesenchymal Transition (EMT). Invasive and metastatic characteristics of carcinoma cells in primary tumors are mediated by EMT. During EMT, the primary tumor cells lose cell-cell adhesion, have increased intercellular separation, and gain an elongated shape with pseudopodia. There is also dysregulation of Polycomb group proteins (such as BMI1, SUZ12, and EZH2), and changes in the expression of microRNA-200 (miR-200) family. In this study, we developed a chemoresistant colorectal cancer cell line (DLD-1-OxR) by exposing DLD-1 colorectal cancer cells to increasing concentrations of oxaliplatin (a chemotherapy drug used for colorectal cancer), and tested for EMT characteristics. We found that DLD-1-OxR exhibited EMT characteristics by morphologic, biochemical and molecular markers. SUZ12, a Polycomb repressive complex 2 subunit, was upregulated in DLD-1-OxR. The miRNA-200 family members that target SUZ12 were downregulated. Drug resistance is an impediment to chemotherapy and understanding the molecular mechanisms of chemoresistance can lead to its reversal and improvement of chemotherapy outcomes.

## INTRODUCTION

Colorectal cancer (CRC) is the third most diagnosed cancer in men and women and the second leading cause of cancer-related death in the USA [1]. Most cancer-related deaths are due to metastasis of tumor cells into vital organs, causing impairment of function and increasing tumor burden [2, 3]. Accumulating evidence suggests that radiotherapy and/or chemotherapy leads to the formation of resistant cells that become metastatic cancer cells [4-6]. The cellular program responsible for the change of epithelial tumors (such as breast, colorectal, pancreatic, ovarian) into metastatic mesenchymal cell types is Epithelial-Mesenchymal Transition (EMT). EMT is a normal process during embryogenesis where epithelial cells lose their characteristics and become motile mesenchymal cells [7]. Several studies show that chemotherapy and radiation treatment are associated with EMT induction, leading to invasive CRC [4, 5, 8].

Tumor metastasis involves multiple steps that lead to migration of tumor cells to distant sites. The process starts by dysregulation of cell adhesion, downregulation of E-cadherin, and upregulation of vimentin and N-cadherin [9]. EMT leads to upregulation of E-cadherin transcriptional repressors such as Snail, Slug, FOXC2, Twist, ZEB1, and ZEB2 [2, 6]. Another hallmark is dysregulation of Polycomb Group (PcG) proteins [10]. PcG proteins are transcriptional repressors involved in cellular differentiation during development and form two major Polycomb repressive complexes (PRC): PRC1 and PRC2 [11, 12]. Several studies show that one subunit of PRC1, BMI1, and two subunits of PRC2, EZH2 and SUZ12, are overexpressed in cancer cells [11, 13]. Overexpression of SUZ12 is implicated in cell proliferation, inhibition of apoptosis, and promotion of cell invasion and metastasis [14, 15].

In addition to PcG protein involvement, deregulation of the microRNA-200 (miR-200) family is implicated in EMT [14, 16]. Micro RNAs (miRNAs) are non-coding RNAs that are 21-23 nucleotides long and regulate gene expression at the post-transcriptional level [17]. A single miRNA can target many genes [17]. The miR-200 family members (miR-200a, -200b, -200c, -141 and -429) regulate EMT by targeting ZEB1 and ZEB2 [16, 18]. Loss of miR-200 leads to increased ZEB1 and ZEB2 levels, which cause the promotion of EMT and downregulation of the gene that encodes E-cadherin, CDH1 [18].

Recent experiments have shown that induction of EMT is associated with the presence of cancer stem cells (CSCs) [7]. These tumor-initiating CSCs have been hypothesized to provide a reservoir of cells that can cause tumor recurrence after therapy [7]. EMT cells with chemoresistance are associated with CSC signature markers, thereby implicating EMT in the generation of stem-like cells [19, 20]. In CSCs, downregulation of miR-200 leads to upregulation of SUZ12, which is implicated in CSC formation and tumor growth [14, 21].

In our study, we tested the hypothesis that CRC chemoresistant cells that have undergone EMT overexpress SUZ12 due to downregulation of miR-200. Since CRC originates in epithelial structures of the large bowel [22], we used a DLD-1 CRC adenocarcinoma cell line to derive a chemoresistant cell line. Oxaliplatin is a commonly used chemotherapeutic agent against CRC [23]. We developed an oxaliplatin-resistant DLD-1 CRC cell line (DLD-1-OxR) which exhibited EMT markers assessed by immunochemical and molecular methods. We found increased SUZ12 expression in DLD-1-OxR compared to the parental DLD-1 cells that correlated with decreased miR-200 (miR-200b and miR-200c) expression.

## RESULTS AND DISCUSSIONS

### DLD-1-OxR exhibited resistance to oxaliplatin, mesenchymal morphological appearance and increased migration

**Figure 1:**
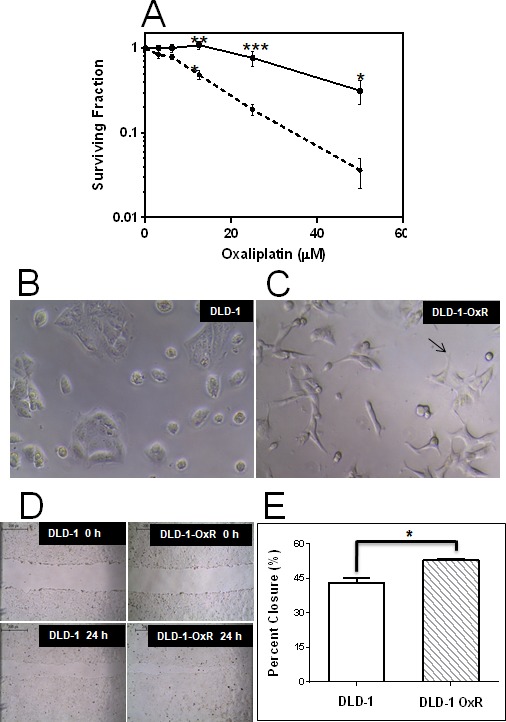
DLD-1-OxR exhibited resistance to oxaliplatin, mesenchymal cell morphology and increased cell migration compared to parental cells **A.** The effect of oxaliplatin on DLD-1 (dashed line) and DLD-1-OxR (solid line) by clonogenic assay. Both DLD-1 and DLD-1-OxR cells were exposed to varying concentrations of oxaliplatin, and surviving colonies were counted. Each data point represents the mean of four independent experiments and three replicates per experiment. Means ± SEM were displayed. * = *P* < 0.05, ** = *P* < 0.01, *** = *P* < 0.001. *n* = 4. $\text{Surviving Fraction}=\frac{\text{No. of colonies formed after treatment}}{\text{No. of cells seeds }\times \text{ PE}}$. Plating efficiency (PE) was calculated as the ratio of the number of colonies formed to the number of cells seeded without treatment. **B.** and **C.** Oxaliplatin-resistant DLD-1-OxR cells exhibited typical mesenchymal phenotype (10x magnification). Cell clusters were evident in parental DLD-1 cells **B.** and absent in DLD-1-OxR cells **C.** Loss of cell clustering was due to loss of cell-cell adhesion by oxaliplatin-resistant cells. Oxaliplatin-resistant cells were spindle-shaped and had pseudopodia (note arrow in C). These morphological changes are consistent with a mesenchymal phenotype. **D.** and **E.** Oxaliplatin-resistant DLD-1-OxR cells exhibited increased cell migration in a wound healing (closure) assay (10x magnification). **D.** Representative images of wound assay results at 0 and 24 hours after scratch. **E.** Percent closure results for DLD-1 and DLD-1-OxR. Means ± SEM results were displayed. * = *P* < 0.05. *n* = 3.

We compared the oxaliplatin-resistant DLD-1-OxR cell line with the parental DLD-1 cell line by clonogenic assay. Chemoresistant cells were maintained at a clinically relevant concentration of 2μM. Cells were plated as single cell suspensions, incubated for 24 hours, and exposed to different concentrations of oxaliplatin. The surviving fraction was plotted against drug concentration. At concentrations above 20μM, DLD-1-OxR cells were significantly resistant to oxaliplatin compared to DLD-1 cells (Figure 1A).

DLD-1-OxR exhibited a different morphological appearance than the parental DLD-1 cells (Figure 1B). The phenotypic changes observed are intercellular separation and increased pseudopodia (Figure 1C). The changes observed are typical of a mesenchymal phenotype.

One of the hallmarks of EMT cells is increased cell movement. For this reason, a cell migration assay was performed using a scratch wound healing assay. Briefly, cells were grown to confluency, and a scratch was made with a pipet tip. Cell migration into the scratch was visualized by photography at time zero and after 24 hours incubation (Figure 1D, 1E). DLD-1-OxR cells exhibited faster migration rates than those of the DLD-1 cells (Figure 1 D, 1E).

### DLD-1-OxR cells exhibited EMT characteristics compared to parental cells

**Figure 2:**
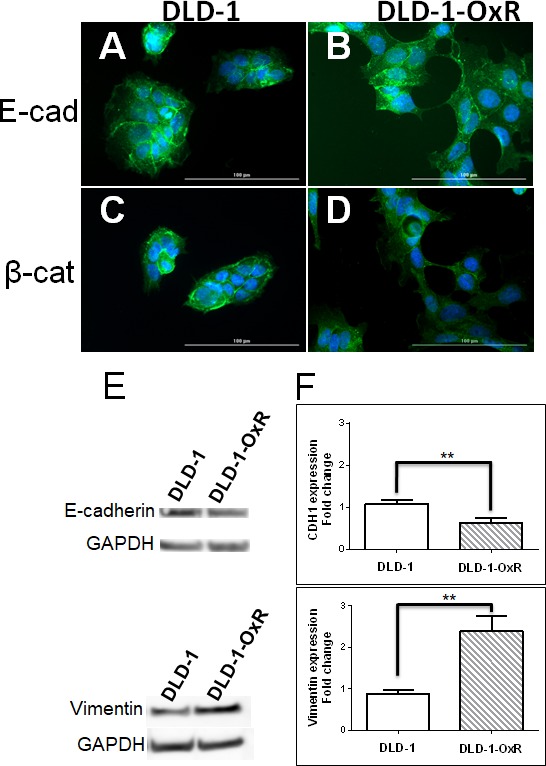
DLD-1-OxR exhibited mesenchymal markers **A.-D.** DLD-1-OxR cells exhibited changes in localization of E-cadherin (E-cad) and β-catenin (β-cat) compared to parental DLD-1 cells as identified by immunofluorescence. The parental cell line showed high amounts of E-cadherin **A.** and β-catenin **C.** in the membrane. Oxaliplatin-resistant cells showed disorganized E-cadherin **B.** and β-catenin **D.** from the membrane location. In DLD-1-OxR, E-cadherin was disorganized and present in the cytoplasm, and β-catenin had translocated to the nucleus. DAPI (blue) was used to counterstain the nucleus. **E.** and **F.** DLD-1-OxR cells exhibited molecular changes consistent with EMT. Cell lysates from DLD-1 and DLD-1-OxR were subjected to Western blot and representative results were displayed **E.**. GAPDH was used as loading control. Compared to DLD-1, decreased levels of E-cadherin and increased levels of vimentin proteins were observed in DLD-1-OxR. Gene expression levels of E-cadherin-encoding CDH1 and vimentin were analyzed by quantitative reverse transcription PCR **F.** Expression levels of E-cadherin were lower and vimentin higher in DLD-1-OxR compared to DLD-1. Means ± SEM were displayed. ** = *p* < 0.01. *n* = 4.

The observed phenotype of DLD-1-OxR cells, based on morphological characteristics and increased cell migration, indicated that the chemoresistant cells had transitioned to a mesenchymal phenotype. A hallmark of EMT is the breakdown of the cytoplasmic cell adhesion complex; E-cadherin and β-catenin are subsequently delocalized from the membrane [4, 7]. Loss of E-cadherin from the membrane disrupts epithelial cell adhesion and leads to release of β-catenin from the intracellular membrane surface. The released β-catenin translocates to the nucleus and, in combination with other transcriptional complexes, leads to changes associated with EMT [4, 7]. Immunofluorescence was done on DLD-1 and DLD-1-OxR for E-cadherin and β-catenin. Loss of E-cadherin and β-catenin from the membrane was observed in DLD-1-OxR cells (Figure 2B, 2D). In DLD-1 cells, both markers were present in the membrane (Figure 2 A, 2C). E-cadherin was disorganized and dispersed throughout the cytoplasm (Figure 2B) and β-catenin was translocated from the membrane to the nucleus (Figure 2D) in DLD-1-OxR cells.

Another phenotype characteristic of EMT is loss of E-cadherin and gain of mesenchymal markers such as vimentin. Western blot analysis showed that E-cadherin expression was lower and vimentin expression was higher in DLD-1-OxR compared to DLD-1 cells (Figure 2E). To confirm that the expression levels of E-cadherin and vimentin were different in DLD-1-OxR, we performed RT-qPCR. E-cadherin gene (CDH1) expression was significantly lower compared to the DLD-1 cells (Figure 2F). On the other hand, gene expression of vimentin was higher than DLD-1 cells (Figure 2F). Both CDH1 downregulation and vimentin upregulation were indicative of cells having undergone EMT.

### Decreased expression of miR-200b/c leads to increased level of its target SUZ12 in DLD-1-OxR cells

**Figure 3:**
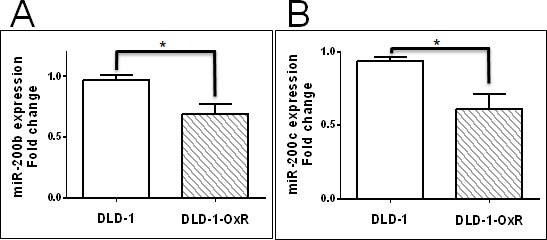
Expression of miR-200b and miR-200c in DLD-1 and DLD-1-OxR cells were decreased compared to DLD-1 parental cells Expression levels of miR-200b **A.** and miR-200c **B.** in DLD-1 and DLD-1-OxR were analyzed by quantitative reverse transcription PCR. Both miR-200b and miR-200c expression levels were decreased in DLD-1-OxR compared to DLD-1. Means ± SEM were displayed. * = *p* < 0.05. *n* = 5.

The drug screening panel of 60 cell lines from the National Cancer Institute was genetically divided into two clusters based on gene signature: mesenchymal and epithelial [24]. By analyzing 207 miRNAs in these cells lines, it was found that expression levels of the miR-200 family predicted whether cancer cells were mesenchymal or epithelial [24]. The miR-200 family consists of five members (miR-200a, -200b, -200c, -141, and -429) expressed in two genomic clusters: one on chromosome 1p36.33 and the other on chromosome 12p12.31 [24]. High expression of miR-200 family members correlated with high expression of E-cadherin, whereas low expression of miR-200 family members correlated with high expression of vimentin. Because miR-200 targets the transcriptional repressors of E-cadherin, expression of miR-200 and E-cadherin was high in epithelial cancers [24]. However, in mesenchymal cancers or those having undergone EMT, expression of vimentin was upregulated, while expression of miR-200 was downregulated [24]. To verify the expression of miR-200 family members, we measured the levels of miR-200b and miR-200c by RT-qPCR. Both miR-200b and miR-200c were downregulated in DLD-1-OxR compared to in DLD-1 (Figure 3A, 3B). This downregulation of miR-200b and miR-200c indicated that the chemoresistant cells had undergone EMT.

**Figure 4:**
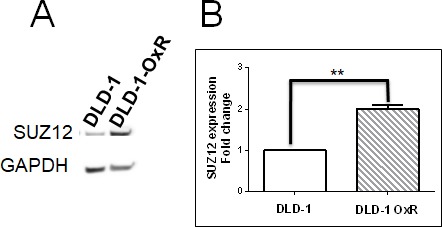
DLD-1-OxR cells overexpressed SUZ12 **A.** Cell lysates from DLD-1 and DLD-1-OxR were subjected to Western blot and representative results displayed. SUZ12 protein levels were increased in DLD-1-OxR compared to DLD-1. GAPDH was used as the loading control. **B.** Expression levels of SUZ12 in DLD-1 and DLD-1-OxR. The expression level of SUZ12 was analyzed by quantitative reverse transcription PCR. The expression level of SUZ12 was higher in DLD-1-OxR compared to DLD-1 parental cells. Means ± SEM were displayed. ** = *p* < 0.01. *n* = 4.

EMT of cancer cells occurs by downregulation of miR-200 family members, and leads to increased expression of transcriptional repressors ZEB1 and ZEB2, causing suppression of CDH1 [24]. Also, miR-200 family members target SUZ12, a subunit of the PRC2 complex [25]. In EMT, downregulated miR-200 leads to high expression of SUZ12. The overexpression of SUZ12 leads to H3-k27 trimethylation at the CDH1 promoter, which represses E-cadherin expression [14]. We tested whether decreased miR-200b and miR-200c correlated with increased SUZ12 in DLD-1-OxR cells. We compared the expression of SUZ12 in DLD-1-OxR and DLD-1 by Western blot. Results showed that SUZ12 expression was increased in DLD-1-OxR compared to DLD-1 cells (Figure 4A). Next, we analyzed SUZ12 mRNA expression by RT-qPCR. The DLD-1-OxR cells exhibited an increase in SUZ12 gene expression compared to DLD-1 (Figure 4B). The results suggested that chemoresistant cells that had undergone EMT downregulate miR-200b/c and upregulate SUZ12 (Figure 4B).

The process of tumor metastasis consists of multiple steps, all of which are required to achieve tumor spreading to different organs [3, 26]. Morphological and molecular changes observed in metastatic cancer cells correlate with those of mesenchymal cell types. EMT is a routine process during embryogenesis; epithelial cells lose adherent characteristics and become more motile like mesenchymal cells [4, 8]. Recently, it was demonstrated in clinical studies and *in vitro* model systems that EMT transition is induced in colorectal cancer cells by radiation and chemotherapy [27, 28]. Accumulating evidence suggests that chemoresistant cancer cells become metastatic by EMT [4, 5, 28, 29].

In the present study, we established and characterized an oxaliplatin-resistant CRC cell line, DLD-1-OxR, that had undergone EMT and exhibited the mesenchymal phenotype morphologically and by molecular markers. DLD-1-OxR showed increased cell migration compared to parental DLD-1 cells. When epithelial cells change to a mesenchymal cell morphology, this transition is associated with changes in expression of molecular markers. During EMT, the amount of surface expression of E-cadherin (CDH1) decreases and mesenchymal-specific marker vimentin increases [4, 7, 8]. Using Western blot technique, we showed that protein expression of E-cadherin decreases, and vimentin increases in DLD-1-OxR. Additionally, CDH1, the gene encoding E-cadherin, was downregulated and vimentin was upregulated compared to DLD-1. Furthermore, DLD-1-OxR showed changes in localization of cellular markers. Immunocytochemistry stain demonstrated that the normally-organized E-cadherin membrane-bound structure became disorganized and dispersed throughout the cytoplasm. Also, β-catenin was observed in the membrane of parental cells, but was translocated to the nucleus in DLD-1-OxR cells.

The chemoresistant cell line overexpressed SUZ12, a PRC2 subunit. SUZ12 is overexpressed in several human cancers and implicated in carcinogenesis [14, 15, 21]. SUZ12 acts as an oncogene by stimulating cell proliferation, blocking apoptosis, and promoting cell invasion and metastasis. MiR-200b/c has been shown to target SUZ12 and CDH1 transcriptional suppressors ZEB1 and ZEB2 [14, 21, 30]. Cancer stem cells with tumor initiating properties were shown to be formed in normal mammary epithelial cells by EMT transcription factors Snail1, Twist1 and ZEB1 [14]. Twist1 alone was able to suppress CSC marker CD24 expression and initiate CSC formation, thus showing the link between EMT and CSC [2]. The miR-200b binding site on the 3’ untranslated region of SUZ12 was highly conserved among species, indicating the importance of SUZ12 regulation by miR-200b [31]. When nontransformed breast epithelial cells were converted by an inducible Src oncogene to the transformed state, a subpopulation of the cells formed CSCs [14]. In these CSC subpopulations, miR-200b was selectively downregulated, and SUZ12 was upregulated [14]. In the present study, we show that downregulation of miR-200b/c and upregulation of SUZ 12 leads to EMT in CRC.

Several studies have shown that EMT is necessary for the metastasis of cancer and occurs in chemoresistant cells [6, 8, 28, 29, 32]. However, recent studies using mouse models showed that EMT is dispensable for metastasis, but is linked to drug resistance [33, 34]. Some of the mechanisms by which EMT augments chemoresistance include downregulation of apoptotic signaling pathways, enhanced drug efflux, and slowed cell proliferation [3, 6, 30, 35]. Drug resistance is a major barrier to successful treatment and a major cause of chemotherapy failure. The propensity of chemoresistant cells to undergo EMT may be a possible survival mechanism. Understanding expression patterns of miRNAs, their targets, and signaling pathways are critical in reversing EMT and the chemoresistant phenotype.

## MATERIALS AND METHODS

### Cell lines and reagents

Human colon cancer cells (DLD-1) were obtained from the American Type Culture Collection (Manassas, VA). The cells were cultured (37°C, 5% CO_2_) in Dulbecco's modified Eagle's medium (DMEM) supplemented with 10% fetal bovine serum, penicillin/streptomycin, glutamine, sodium pyruvate, and HEPES buffer. DMEM and culture medium supplements were purchased from Hyclone (Logan, UT). Oxaliplatin (Sigma, St. Louis, MO) stock solution 50mM was prepared in DMSO. All the antibodies were obtained from Cell Signaling Technology (Danvers, MA).

### Development of DLD-1-OxR cell line

To develop an oxaliplatin-resistant cell line, DLD-1 cells were grown in increasing concentrations of oxaliplatin from low (0.1 μM) to high (2μM) with media replacement every 2 days. After five passages, individual clones were selected by single cell cloning using limiting dilution. Single colonies having mesenchymal morphology were selected and propagated in oxaliplatin (2μM)-containing media. Comparison of resistance to oxaliplatin between the developed DLD-1-OxR and parental cells was assessed by clonogenic assay.

### Clonogenic assay

The sensitivity of DLD-1 and DLD-1-OxR cells to oxaliplatin was measured by clonogenic survival assay [36]. Cells were plated in 6-well plates (300 cells/well) and treated with oxaliplatin (3.125μM to 50μM) or with media containing vehicle control (DMSO). Each concentration was done in triplicate, and four independent experiments were performed. The surviving cells were allowed to form colonies for 8-12 days, and were then fixed with methanol and stained with 1% crystal violet. The colonies with 50 or more cells were counted using Cytation 3 Cell Imaging Multi Mode Reader (Biotek, Winooski, VT). Since only a fraction of the seeded cells retain the capacity to form colonies, we calculated the plating efficiency of the untreated DLD-1 and DLD-1-OxR cells. Plating efficiency (PE) was calculated as the ratio of the number of colonies formed to the number of cells seeded without treatment. The surviving fraction was calculated as the surviving colony fraction of the treatment plates (colonies counted/total cells seeded) divided by the PE.

$$\text{Surviving Fraction}=\frac{\text{No. of colonies formed after treatment}}{\text{No. of cells seeds }\times \text{ PE}}$$

### Migration assay/wound healing assay

DLD-1 and DLD-1-OxR cells were grown to 100% confluency. Perpendicular lines were scratched in the monolayer, the cells were washed, and fresh media was added. Cell migration into the scratch was identified by photography at time zero and after 24 hour incubation. Area of closure was measured from images acquired using Image J software (http://rsb.info.nih.gov/ij/). Percent closure was calculated using the following formula:

$$\text{Percent closure}=\frac{\text{Area of original wound}-\text{Area of wound during healing}}{\text{Area of original wound}}\times 100\%$$

### Immunocytochemistry

DLD-1 and DLD-1-OxR cells were grown on coverslips to 50% confluency. Cells were fixed with ice cold methanol followed by 4% paraformaldehyde. The coverslips were rinsed with PBS and blocked with PBS containing 5% normal goat serum and 0.3% Triton X-100. The coverslips were incubated overnight with rabbit monoclonal primary antibodies against E-cadherin and β-catenin (Cell Signaling Technology, Danvers, MA). The unbound primary antibodies were removed by washing with PBS. The coverslips with the cells were then incubated with Alexa Fluor 488 fluorophore-conjugated secondary antibody (Invitrogen, Carlsbad, CA), and the unbound secondary antibodies were then removed by washing with PBS. The coverslips were stained with DAPI (Sigma, St. Louis, MO) and mounted on a glass slide with Prolong Gold Antifade reagent (ThermoFisher, Waltham, MA). The immunostained cells were visualized by fluorescence microscope.

### Western blot

DLD-1 and DLD-1-OxR cells were solubilized in lysis buffer (50mM Tris, 100mM NaCl, 2.5mM EDTA, 1% TritonX-100, 1% Nonidet P-40, 2.5mM sodium orthovanadate, 25μg/ml aprotinin, 25μg/ml leupeptin, 25μg/ml pepstatin A, and 1mM phenylmethylsulfonyl fluoride) and centrifuged (10,000g, 15 min). The supernatant was removed, and protein concentration was determined by the BCA method (Pierce Biotechnology, Rockford, IL). Cell lysates containing 20μg of protein were separated by 4-12% bis-Tris gel (Invitrogen, Carlsbad, CA) electrophoresis, after which proteins were transferred electrophoretically onto a nitrocellulose membrane (Pierce Biotechnology, Rockford, IL). The membrane was blocked using blocking buffer (TBST: 20mM Tris, pH 7.6, 100μM NaCl, 0.1% Tween-20, 5% nonfat dry milk) and incubated for 1 hour at room temperature with gentle agitation. After blocking, the membrane was washed with TBST and incubated with primary antibody (E-cadherin, vimentin or SUZ12) in TBST blocking buffer overnight at 4°C. Primary antibody was then removed, the membrane was washed three times with TBST and incubated with horseradish peroxidase-labeled secondary antibody for 1 hour at room temperature. Immunoreactive bands were visualized by a chemiluminescent detection system (Pierce Biotechnology, Rockford, IL). GAPDH was used as the loading control.

### RNA isolation

Cell pellets were lysed with Trizol (Zymo Research, Irvine, CA), and total RNA was purified using the Direct-Zol RNA kit (Zymo Research, Irvine, CA) following the manufacturer's protocol. RNA concentration was determined spectrophotometrically, and the integrity was checked using gel electrophoresis.

### Real-time quantitative RT-PCR

Total RNA (2μg) was used to generate cDNA using the High-Capacity cDNA Reverse Transcription kit (Applied Biosystems, Foster City, CA). The mRNA expression levels were measured using TaqMan Gene Expression Assay (Applied Biosystems, Foster City, CA) on a 7500 Real-time PCR System (Applied Biosystems, Foster City, CA). The β2 microglobulin (β2M) was used as endogenous control. TaqMan gene expression assays used were Hs00185584_m1 (vimentin), Hs01023894_m1 (CDH1), HS00248742_m1 (SUZ12), and Hs00984230_m1 (β2M). For each sample, the Mean Ct value was calculated from three technical replicates and β2-M was used for normalization with fold changes calculated as 2^−ΔΔCT^ [37].

Expression levels of miR-200b and miR-200c were analyzed by TaqMan quantitative real-time PCR (Applied Biosystems, Foster City, CA). Briefly, the method was as follows: in the first step, RT-PCR (Applied Biosystems, Foster City, CA), with a stem-loop specific primer was used to generate cDNA from total RNA. In the second step, the cDNA was amplified by TaqMan micro RNA assay using Universal PCR Master Mix. All reaction conditions were according to manufacturer's recommendation. The small RNA (snoRNA) RNU48 was used as endogenous control to normalize the data. The delta-delta Ct method was used to calculate fold-change. The TaqMan gene expression assays used were 000505 (hsa-miR-200c), 002251 (hsa-miR-200b), and 001006 (RNU48).

### Statistics

Comparisons of oxaliplatin sensitivity by clonogenic assay were performed using one-way analysis of variance (ANOVA) with Newman-Keuls post hoc test. Statistical comparisons between parental and chemoresistant cells for RT-qPCR and wound healing assays were by Student's t test. All experimental data were reported as mean ± SEM (standard error of mean), and at least three independent experiments were performed. *P* < 0.05 was considered statistically significant unless otherwise indicated.
